# Bacterial contaminants in stored blood and blood products at Zomba Central Hospital Blood Bank: assessing the possible risk of post-transfusion sepsis in a resource-limited setting

**DOI:** 10.11604/pamj.2024.49.42.45119

**Published:** 2024-10-16

**Authors:** Eshanie Alfred Office, Thomas Claydon Salimu

**Affiliations:** 1 Ministry of Health, Zomba Central Hospital, Laboratory Department, Zomba, Malawi,; 2 Mzuzu University, Faculty of Health Sciences, Department of Biomedical Sciences, Luwinga, Mzuzu, Malawi

**Keywords:** Bacterial contamination, sepsis, transfusion-transmitted infection, blood transfusion

## Abstract

**Introduction:**

National Blood Transfusion Services have done a commendable job in reducing transfusion-related fatalities from viruses, syphilis, and malaria through the vigilant screening of blood donors and donated blood. Bacterial contamination of blood products remains the commonest cause of transfusion-associated fatalities, but it remains unaddressed in resource-limited countries. Up-to-date knowledge of the prevalence and causes of bacterial contamination of blood products is necessary to ensure safe blood transfusion. This study investigated the rate and spectrum of bacterial contaminants in stored blood products at the Zomba Central Hospital from October to November 2022.

**Methods:**

in this cross-sectional study, a total of 115 blood products (whole blood, packed red blood cells, and platelets) were randomly and aseptically collected into Tryptic Soy Broth and then incubated for 7 days. After overnight incubation, all samples were subcultured onto BA, CA, and MAC. Colony morphology, gram staining, and biochemical tests were used for identification. Descriptive, correlation, and regression statistics were used, and results with p ≤ 0.05 were considered significant.

**Results:**

out of the 115 samples, 21 (18.3%, CI: 11.7%-26.6%) were contaminated with various gram-positive bacteria. The contaminants were Bacillus spp (33.33%), Listeria spp (33.33%), coagulase-negative Staphylococcus (19.05%), S. aureus (9.52%), and Enterococcus sp (4.76%). 90.5% of all the contaminated products had exceeded 2 storage weeks.

**Conclusion:**

bacterial contamination of stored blood products is common at the study site presenting a significant risk of post-transfusion sepsis to the recipients. This study emphasizes the need to implement hemovigilance projects aimed at reducing bacterially contaminated blood products.

## Introduction

Blood transfusion is a medical practice that has served an important role in saving lives and improving the quality of life in a variety of clinical conditions for the past few centuries [1]. However, whole blood and blood components for transfusion can be a source of transfusion-transmitted infections (TTIs) from viral, bacterial, and parasitic pathological agents [2,3]. Recently, much emphasis has been directed towards minimizing the risk of transmission of viruses, *Treponema pallidum*, and *Plasmodium* species through blood transfusion. Because of the high prevalence of immunosuppressive diseases like HIV in Africa, the low but definite risk of bacterial contamination continues to pose a significant threat to the safety of blood transfusion [4-6].

Bacterial infection through blood may lead to complications like sepsis, pneumonia, abscesses, wound infection, meningitis, haemolysis, empyema, urinary tract infection, and fever [7,8]. In developed countries, 57% of all TTIs and a mortality of 16% from all TTIs have been attributed to bacterial contamination of blood and blood components [9]. Even after taking various precautions during blood collection and processing, bacterial contamination still takes place through various endogenous and exogenous means [10]. Bacterial inoculation into blood bags may result from insufficient disinfection of the venepuncture site, already existing asymptomatic bacteraemia from the donor, contamination from the environment, or handling blood during blood component preparation [11].

Another factor that is overlooked is the storage conditions of these blood products. With the frequent power outages in countries like Malawi, there are likely to be temperature fluctuations in the blood banks which may facilitate the proliferation of bacteria. Most of the preventive actions currently implemented by developed countries are not yet accessible in developing countries including Malawi [12]. For developed countries like the United States of America, France, and the United Kingdom, studies report that bacterial contamination of donated blood is at 0.2%, 0.1%, and 0.15%, respectively [10]. The numbers are higher in African studies, however, with the rates estimated to be between 8-18% according to Onchaga *et al*. [13]. The organisms most commonly isolated from contaminated blood and blood components include normal flora from the skin and bacterial contaminants from the environment. Predominantly, gram-positive organisms, including *Bacillus* species, *Streptococcus* species, *Staphylococcus* species, and gram-negatives like *Yersinia enterocolitica, Pseudomonas fluorescens, Pseudomonas aeruginosa, Salmonella* species, and *Escherichia coli* have all been implicated [9,13,14].

Bacterial contamination is a leading cause of transfusion-related deaths after ABO-mismatch [15]. Various improvements in donor screening and pre-transfusion testing have helped in deflating transfusion-transmitted viral infections to less than one in a million blood transfusions [16,17]. In contrast, there has been at least one culture-confirmed post-transfusion sepsis case out of 100,000 recipients with a fatality of 1 in 500,000 recipients [13]. Procedures like leukocyte depletion, diversion methods, and pre-donation sampling can help to minimize transfusion-related bacterial infections from contaminated blood and blood products [18]. However, most of these methods are not yet accessible in developing countries including Malawi. Though sporadic, the data collected within sub-Saharan Africa estimated bacterial contamination ranging from 8.8% to 17.5% [13]. With the rapid emergence of antimicrobial-resistant bacteria, the prevention of septicaemia by providing safe blood and blood products can reduce mortalities if taken seriously. Although transfusion-associated bacterial sepsis has been given insufficient acknowledgement in Malawi, it remains a serious public health matter.

This study, which was conducted at Zomba Central Hospital (ZCH), therefore, assessed the bacterial contamination of stored blood and blood products collected and prepared by the Malawi Blood Transfusion Services. We also investigated the relationship between storage time and the frequency of bacterial contamination.

## Methods

**Study design and setting:** this study used a laboratory-based cross-sectional study design with a quantitative application to demonstrate the bacterial contamination of blood and blood products. It was conducted at ZCH, one of the referral centres in Malawi. We used the Krejcie and Morgan formula at a 95% level of confidence with a precision of 5% to calculate the sample size. A total of 115 samples were collected from whole blood, packed red cell concentrates and platelet concentrates. A simple random sampling technique was employed to come up with the study participants. Fresh frozen plasma was not sampled as it could not be refrozen after thawing. For this project, data collection lasted 5 weeks from October to November 2022.

**Figure 1:**
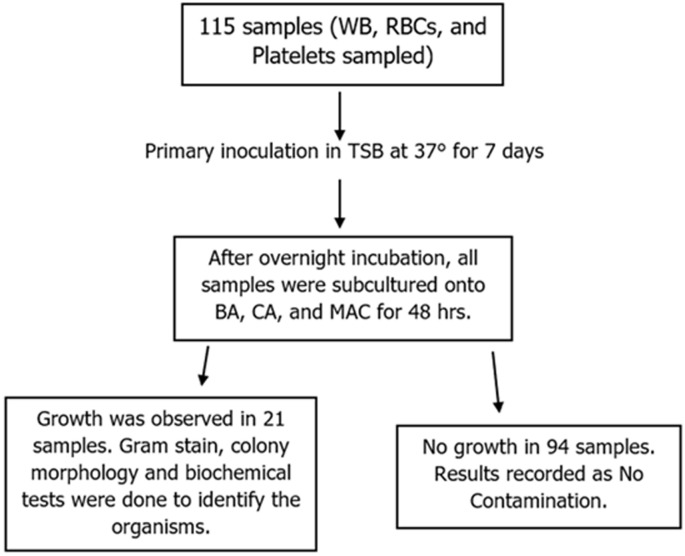
sample flowchart for the procedure for processing the samples at Zomba Central Hospital Blood Bank and Microbiology, from October to November 2022

**Sample collection and culturing:** samples were collected from the portions at the end of the satellite tube linings with at least 1ml collected per pint. After thoroughly disinfecting the tube portions with 70% isopropyl and 2% tincture of iodine, a pair of sterilized scissors was used to cut the two ends of the portions. Then the blood was left to flow into the pre-enrichment culture Tryptic Soy Broth (TSB) media (Figure 1). To prevent contamination of the samples, the procedure was conducted in a well-disinfected biosafety cabinet. All broth suspensions were incubated aerobically at 37ºC. After overnight incubation, a loopful of every sample in the enrichment media was then subcultured onto blood agar, chocolate agar, and MacConkey agar according to the standard operating procedures. Chocolate agar plates were incubated anaerobically.

**Identification of bacterial isolates:** for species identification, colonial morphology description was employed followed by a gram-stain test and necessary standard biochemical tests for further identification of the bacterial isolates.

**Data analysis:** data recorded on Microsoft Excel 2016 was cleaned and analyzed by Statistical Package for Social Sciences (SPSS) software version 22.0 (IBM, USA). Descriptive statistics, linear regression, correlation statistics, and two-sample t-tests were computed to analyze the findings of the study. Charts and tables were used to present the distribution of bacterial contaminants. Correlation and regression statistics including the two-sample t-test and Phi and Cramer’s V correlation statistics were calculated to analyze the relationship between the blood type, storage days, and the frequency of contamination. There was no missing data in this project. Findings were regarded as significant if p≤0.05. Frequencies and proportions of a 95% confidence interval were also used to present the data in tables and charts.

**Ethical considerations:** ethical approval was acquired from the Mzuzu University Research Review Committee under the Health Sciences Faculty Research Committee (Ethical Clearance number: FOHS/REC/21/202). An approval letter from the ZCH Research Committee was also obtained for permission to conduct the study at the ZCH Laboratory.

## Results

Out of the 115 eligible blood products, the majority of the units were whole blood (n=57, 49.6%), followed by packed red cell units (n=52, 45.2%) and platelet units (n=6, 5.2%) (Table 1). There were 61 blood products of blood group O RhD positive representing 53.0%, then 25 (21.7%) units of blood group A RhD positive, 24 (20.9%) units of blood group B RhD positive, and 5 (4.3%) units of blood group AB RhD positive. The sample did not contain RhD-negative products, as they are scarce in the blood bank and only available after an emergency order. The storage duration of the blood products ranged from 4 to 40 days. Additionally, the average storage days of the sampled blood products was 15.10 with a standard deviation of 8.19 days.

**Table 1:** characteristics of the sampled blood products from the Zomba Central Hospital Blood Bank, from October to November 2022 (N=115)

Description	Number	%
**Type of blood components**		
Whole blood N (%)	57	49.6
Packed red cells N (%)	52	45.2
Platelets N (%)	6	5.2
**Blood groups of blood components**		
A Rh D positive N (%)	25	21.7
B Rh D positive N (%)	24	20.9
AB Rh D positive N (%)	5	4.3
O Rh D positive N (%)	61	53
**Statistics for storage days**		
Minimum	4	-
Maximum	40	-
Mean	15.1	-
Standard deviation	8.19	-

**Figure 2:**
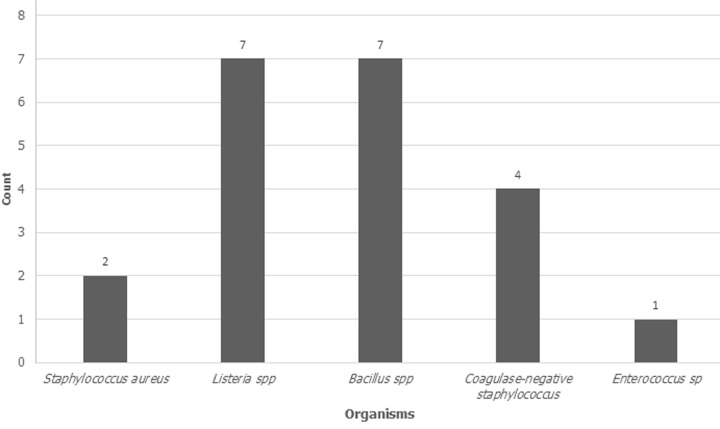
bar chart of the distribution of the isolated organisms from Zomba Central Hospital Blood Bank, from October to November 2022 (N=21)

The overall prevalence of bacterial contamination was 18.3% (CI: 11.7%-26.6%) as 21 of the 115 blood product samples were found to be contaminated with bacteria, while 94 (81.7%) showed no bacterial growth after 48 hours of incubation (Figure 1). In this study, all 21 organisms isolated from the blood products were gram-positive bacteria. The majority of the isolates included *Bacillus spp* (33.33%) and *Listeria spp* (33.33%) (n=7) while 19.05% were Coagulase-negative *Staphylococcus* (n=4), followed by 2 *S. aureus* isolates (9.52%) and 1 *Enterococcus sp* bacteria representing 4.76% (Figure 2). The possible sources of the bacterial contaminants include donor skin, donor blood, and the environment mostly the soil and water. Of the isolated organisms, 71.36% are known environmental bacteria, and 38.09% are skin-normal flora. Gram-negative species were not isolated in this study. Platelets had a higher contamination rate (33.3%, n=2/6) as compared to the rest of the products. Of the 52 packed RBCs, 11 were contaminated, as well as 8/57 of whole blood units. According to Phi and Cramer´s V correlation statistics, there is no significant association between the type of blood product and the levels of bacterial contamination (p=0.389).

**Figure 3:**
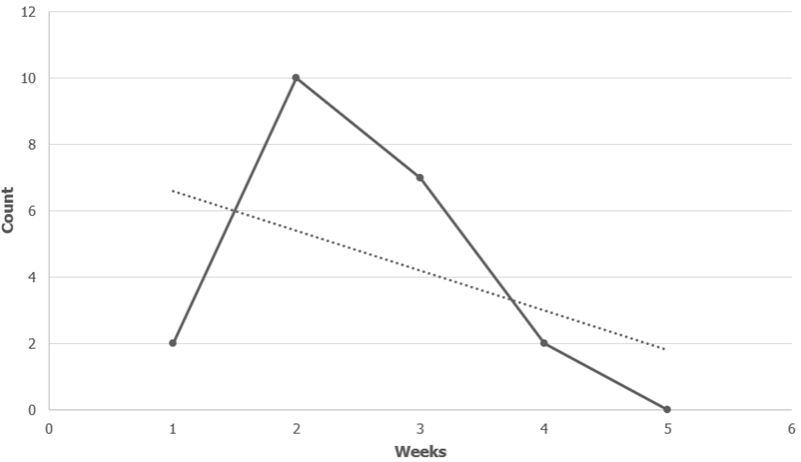
line chart of the frequency of bacterial contamination and the storage weeks at the Zomba Central Hospital Blood Bank

Out of the 21 contaminants isolated, the majority were from the blood group O RhD positive (n=12). Blood group A RhD positive had 4 contaminants isolated. Three and 2 contaminants were isolated from blood groups B RhD positive and AB RhD positive respectively. There was no significant association between the blood group and the bacterial contamination of the blood products (p=0.516). There was no significant difference (p=0.567) in the means of the storage days between the blood products that were contaminated and those that were not contaminated after using the two-sample t-test. The blood units with bacterial contamination had an average storage period of 14.18 days as compared to those without contamination (mean=15.32) (Table 2). The highest rate of bacterial growth was observed in blood products with 2 weeks of storage (n=10). Storage week 3 registered 7 contaminants, while weeks 1 and 4 had 2 contaminants each. All the blood products beyond 4 storage weeks had no bacterial contamination. About 90.5% of the contaminated samples had more than 2 weeks of storage. Linear regression statistics (R= -0.457, CI: -0.591, -0.299) calculated to analyze the relationship between the storage duration and the number of contaminants isolated revealed no significant association between the two variables. However, there was a declining trend observed on the line chart between the frequency of contamination and the storage weeks (Figure 3).

**Table 2:** summary of descriptive statistics for storage days of blood products and the frequency of contamination at Zomba Central Hospital Blood Bank, from October to November 2022

Culture results	Mean (days)	SD	Median
No growth	15.32	8.648	12.00
Growth	14.18	5.949	14.00
Total	15.10	8.190	12.00

## Discussion

**Prevalence of bacterial contaminants in blood and blood products:** in this study, the prevalence of bacterial contamination of blood and its components was 18.3% (n=21/115). This finding is marginally higher but in accordance with studies done in Egypt (17.9%) [19], and Ghana (16.5%) [12]. This prevalence, however, is significantly higher when compared to studies from high-income countries like the United States of America (0.2%), the United Kingdom (0.15%) and France (0.1%) [10]. The wide difference could be because low-income countries like Malawi do not use various preventive implementations like diversion methods, inactivation of pathogens using ultraviolet rays, pre-donation sampling, double disinfection of the phlebotomy site, and the lack of national hemovigilance programmes. Tsegaye *et al*. [20] compared diverging and non-diverging blood collection methods and reported that diverting the first 30-40 ml of blood during collection reduced the bacterial contamination rate by 5.8% in their study. It is also important to note that there were frequent power outages and a lack of backup power in September and October. As a consequence, the blood products were exposed to temperatures greater than 8°C for considerable periods. This might have contributed to the high prevalence of bacterial contaminants, as the bacteria would have been able to survive and proliferate for longer periods.

**Bacteria species isolated in the blood and blood products:** the majority of the isolated organisms were both *Bacillus spp* and *Listeria spp* (33.3%, n=7) followed by coagulase-negative *Staphylococcus* (19.05%, n=4), *S. aureus* (9.52%, n=2) and *Enterococcus sp* (4.76%, n=1). Out of these isolates, only *S. aureus* could have originated from donor blood. This could suggest that healthcare workers are vigilant in the comprehensive screening and recruitment of donors. However, the high prevalence of gram-positive bacteria indicates poor scrubbing of the phlebotomy site during blood collection. Similarly, studies conducted in Uganda and India did not report gram-negative bacteria in their findings [1,2]. In most of the studies that reported gram-negative bacteria as well, gram-positives still had the highest prevalence among the isolates. For instance, from the isolated bacteria, 80%, 77.8%, 82.4% 88.9%, 56.2%, and 79% were gram-positives [6,10,12,13,15,18,20]. These findings are in line with my findings and point towards improper disinfection of the venepuncture site as the commonest source of bacterial contamination of blood and blood components. Nevertheless, the predominant isolation of gram-positive bacteria does not reduce the possibility and severity of post-transfusion sepsis as *Staphylococcus spp* and *Bacillus spp* have been reported to cause the majority of the cases [4,21].

Most of the isolated bacteria are commonly found on the human skin, in the blood, as well as in the environment. *Staphylococcus spp* are normal flora of the skin but *S. aureus* has also been isolated in many cases of bacteraemia [21]. *Bacillus, Enterococcus* and *Listeria spp* are readily found in water and soil. Therefore, the possible sources of the organisms isolated in this study may have included dust within blood donation areas, contaminated cold chain boxes, poor skin disinfection during phlebotomy, as well as donor-asymptomatic bacteraemia. The high prevalence of bacterial contaminants found in this study suggests that blood transfusion in Malawi poses a great risk of transfusion-related bacteraemia. However, since gram-negative were not isolated, the infections from the isolated gram-positive bacteria may not be very severe to immunocompetent individuals. Nevertheless, with the current emergence of antibiotic-resistant bacteria, the increased rates of HIV infection, and non-communicable diseases that lead to immunodeficiency, there are reports of severe cases of sepsis and its complications caused by some of the gram-positive species isolated in this study [21-25]. Whilst bacteraemia from *Enterococcus spp* is extremely rare, it has also been reported to have a high mortality rate when treatment is delayed [26]. Therefore, the gram-positive bacteria isolated in this dissertation still pose a great risk to the safety of blood transfusion in Malawi.

**Distribution of contamination among blood and blood products:** in this study, packed RBCs were the most contaminated blood product with 11 (52%) units contaminated followed by whole blood (n=8, 38%) and platelets (n=2, 10%). These findings were not in agreement with the findings by Gupta *et al*. [14] and Makuni *et al*. [9]. However, the results are biased as the blood products sampled were not of equal proportions. Tsegaye *et al*. [20] conducted a similar study but with equal samples of whole blood, packed RBCs, platelets, and fresh frozen plasma. The findings revealed that whole blood and packed RBCs had a higher bacterial contamination rate than platelets. The correlation statistics calculated showed that there was no significant relationship between the type of blood products and the rate of bacterial contamination (p=0.389). Another study with a larger sample size and equal proportions of blood products needs to be conducted to further assess the difference in the risk of post-transfusion sepsis from different blood products. Although platelets have been reported to have the highest risk of bacterial contamination given their storage conditions among the blood products, this study suggests that all blood products have an equal probability of causing transfusion-related sepsis. There was no significant association between the blood group and the bacterial contamination of the blood products (p=0.516). As opposed to these findings, various studies reported a high rate of contamination for blood group O RhD positive [2,14,20].

**The storage duration and the frequency of bacterial contamination:** literature suggests that bacteria require a period of proliferation in storage before they can be detected and that most of the contaminants are isolated within the first week of storage. This is because the survival and proliferation of bacteria are reduced once the organism enters the blood bag due to proper cold chain storage and limited storage of the blood and blood products. On the contrary, the present study revealed that 90.5% of the contaminated blood and blood products had exceeded 2 weeks of storage. Our results are not in agreement with the study conducted in India where 76.46% of the contaminated units had less than 1 week of storage [14]. Agzie *et al*. [10] also reported that blood units from 0 to 3 days of storage period had the highest contamination rate. A study conducted by Heroes *et al*. [6], states that bacterial contamination was higher in older blood and blood products than the products although the days and weeks were not specified. The reasons for the variations cannot be ascertained from this study. It is important to account, however, that ZCH Laboratory had a few challenges that may help to explain these findings. It was noted that the refrigerator used to store these units was frequently opened during working hours, which might have compromised the cold chain conditions. In addition, there were frequent power failures coupled with the lack of backup power from the hospital generator due to the fuel crisis in the country at the time. All these could have contributed to the longevity of the bacteria in the blood products as they were exposed to temperatures higher than 8°C for long periods. There seemed to be a decreasing trend in the rate of bacterial contamination as the storage weeks increased, as portrayed by Figure 3, indicating that even with poor storage conditions bacteria cannot survive past a specific period in storage. Nevertheless, there was no significant relationship (p>0.05) between the storage period and the rate of bacterial contamination of blood products. A study from Ethiopia had a similar conclusion [2]. Thus, we can hypothesize that the rate of bacterial contamination does not only depend on the storage period but also on the conditions in which the blood products are kept. Ambient, as well as refrigeration temperatures must be constantly monitored with well-calibrated thermometers to ensure proper room and cold chain conditions to reduce the survival and proliferation of bacteria in various blood components.

**Limitations:** since the study only included blood products from one blood donation centre (Blantyre), the findings may not be used to represent the rest of the blood collection centres from Balaka, Lilongwe, and Mzuzu. The study also did not include follow-up of blood recipients for clinical outcomes to check if they developed any kind of septicaemia. Finally, the study was not able to establish the source of the bacterial contaminants, therefore the possible sources elaborated in this report are purely based on commonly established habitats of the isolated bacteria.

## Conclusion

This study revealed that the prevalence of bacterial contamination of stored blood and blood products at ZCH was higher than in various studies conducted in other countries. All isolated organisms were gram-positive hinting at poor disinfection techniques at the phlebotomy site during the blood donation process. *Bacillus* and *Listeria spp* were the most isolated bacteria with coagulase-negative *Staphylococcus, Enterococcus sp* and *S. aureus* also identified. There was no significant relationship between the rate of bacterial contamination of the blood products and the type of blood product, the blood group as well as the storage period (p>0.05). In conclusion, there is a need for serious coordination between the Ministry of Health, the Malawi Blood Transfusion Services (MBTS), and all stakeholders in the blood transfusion process to counter this silent threat. The establishment of a national hemovigilance programme is highly recommended, which can promote the introduction of blood donation bags with diversion pouches and periodic training of healthcare workers on the blood transfusion process including awareness of transfusion-related sepsis. Lastly, there is a need to enforce improved donor arm disinfection using 70% isopropyl alcohol and then 2% chlorhexidine as recommended by the National Health Service in England.

### What is known about this topic

- The presence of bacteria in blood and blood products threatens the safety of blood transfusion and is the major cause of transfusion-related fatalities second to ABO-incompatibility;
- Previous studies in resource-limited countries have reported a high prevalence of bacterial contaminants, unlike reports from developed countries like the UK and the USA;
- No articles have been published in Malawi about the prevalence of bacterial contaminants in blood products.

### What this study adds

- The study assesses the safety of blood transfusion at Zomba Central Hospital, a referral hospital in Malawi;
- Reporting on the risk of post-transfusion sepsis in Malawi provides an important context for the Malawi Blood Transfusion Sciences policymakers;
- The paper also provides possible recommendations to counter the threat of post-transfusion sepsis.
